# Cryogenic Electron Microscopy of Extracellular Vesicles from Temozolomide-Treated Glioblastoma Cells Reveals Great Morphological Heterogeneity

**DOI:** 10.3390/nano16110685

**Published:** 2026-06-01

**Authors:** Mariana Karimova, Giordana Ponziani, Diana Vardanyan, Andrea Alfieri, Maurizio Zuccotti, Stefano Tacconi, Luciana Dini

**Affiliations:** 1 Department of Biology and Biotechnologies Charles Darwin, The Sapienza University of Rome, 00185 Rome, Italyponziani.giordana@gmail.com (G.P.);; 2 Centro Grandi Strumenti, University of Pavia, 27100 Pavia, Italy; 3 Department of Biology and Biotechnology “Lazzaro Spallanzani”, University of Pavia, 27100 Pavia, Italy

**Keywords:** cryo-EM, extracellular vesicles, glioblastoma multiforme, temozolomide

## Abstract

Extracellular vesicles (EVs) are attracting considerable interest due to their important role in cell signaling. However, their nanosized scale, complexity, and heterogeneity make even morphological characterization challenging. Only with the recent advances in cryogenic electron microscopy (cryo-EM), together with the increasing availability of cryo-electron microscopes, has it become possible to visualize the native structure of EVs. In this study we performed an in-depth cryo-EM analysis of EVs derived from four glioblastoma multiforme (GBM) cell lines (U87MG, U373MG, U251MG, and T98G), highlighting the morphological changes induced by temozolomide (TMZ) chemotherapeutic treatment. Size, shape, circularity, concentricity, membrane thickness, and electron density of EVs were analyzed. The key characteristic revealed by cryo-EM was that EVs can be enclosed not only by one membrane bilayer, but also by two or more bilayers (double-layered vesicles, DVs; and multilayered vesicles, MVs). Overall, TMZ treatment substantially modified both the morphology and production of EVs, decreasing the percentage of single-layered vesicles (SVs) while increasing that of DVs and MVs, as well as reducing the electron density of the EV cargo. Morphometric and morphological information can shed light on the contribution of EVs to tumor progression, metabolism, drug resistance, and immune evasion.

## 1. Introduction

Extracellular vesicles (EVs) are nano- and micro-sized membrane-enclosed particles that are released by almost all types of cells under physiological and pathological conditions. The evolutionary conservation of EV release from bacteria to plants and humans underlines their important role in cell-to-cell communication [1]. According to MISEV2023 guidelines, EVs are classified by size into small EVs (<200 nm) and large EVs (>200 nm), as well as by their biogenesis into exosomes and ectosomes. Exosomes are produced through the endosomal pathway by inward budding of membranes in maturating multivesicular bodies (MVBs), where they form intraluminal vesicles (ILVs). The latter are secreted by the fusion of MVBs with the plasma membrane. Ectosomes are formed through the outward budding of the cellular membrane [2,3]. EVs carry various bioactive molecules such as proteins, nucleic acids, and lipids. The composition of EVs depends on the physiological state of the cells, which in turn changes the effect on recipient cells [4,5].

Glioblastoma multiforme (GBM)-derived EVs are known to alter the tumor microenvironment (TME) to favor cancer progression, survival, and resistance to therapy [6]. Glioblastoma is classified as a grade IV glioma, and it is notorious for its heterogeneity and aggressiveness. Despite surgery, radio- and chemotherapy, the survival of patients diagnosed with GBM remains approximately 15 months, making it the most aggressive type of brain cancer in adults [7]. The ineffectiveness of current treatment, including the most common chemotherapeutic drug temozolomide (TMZ), highlights the importance of targeting the pro-tumorigenic TME and stimulates research into the role of EVs. Chemotherapy affects not only targeted cells but also causes changes in the cargo and function of EVs [8]. TMZ treatment of glioma stem cells (GSCs) resulted in elevated levels of adhesion proteins in EV cargo, thus supporting pro-tumoral signaling [9]. Increased expression of heat shock proteins in GBM cell lines and EV cargo following TMZ treatment may contribute to chemotherapy resistance [10]. Moreover, TMZ treatment can lead to elevated levels of transcripts related to TMZ-resistance, as well as an increase in EV release in some resistant GSC cell lines [11]. However, the effect of TMZ on the composition of EVs remains insufficiently studied. Therefore, characterization of EVs using novel morphological and molecular methods can shed light on new ways to manage GBM.

Phenotypic characterization of EVs is the first and one of the most important steps in EV research, which above all provides crucial evidence of the specificity of isolation, the purity, and the integrity of EVs. Cryogenic electron microscopy (cryo-EM) has become an indispensable tool in EV research [12]. It is the only method that allows the visualization of the native structure of EVs. Unlike conventional transmission or scanning electron microscopy (TEM, SEM), which require fixation or usage of contrasting chemicals that alter the morphology of EVs, cryo-EM utilizes only plunge-freezing to preserve the EVs in their most native state [13,14]. The use of cryo-EM changed our perception of EVs as vesicles surrounded by one membrane bilayer and revealed the presence of more complex structures, so-called multilayered or multilamellar EVs [15,16,17].

In this study, we characterized the morphology, structure, and heterogeneity of GBM-derived EVs using cryo-EM and determined how TMZ treatment altered these parameters. Specifically, we investigated the size and morphology of EVs, including single-layered vesicles (SVs), double-layered vesicles (DVs), and multilayered vesicles (MVs), as well as their circularity, concentricity, membrane thickness, and electron density across multiple GBM cell lines (U87MG, U373MG, U251MG, and T98G). By integrating these parameters, this work seeks to provide a deeper understanding of EV structural diversity and to elucidate treatment-associated alterations that may have functional relevance. In addition to revealing the morphological heterogeneity of EVs, an insight into their biogenesis and the role of different EV subtypes can be provided.

## 2. Materials and Methods

### 2.1. Cell Culture

Human glioblastoma cell lines (U87MG, U373MG, U251MG, and T98G) were cultured in Dulbecco’s Modified Eagle Medium (DMEM) 4.5 g/L glucose (Aurogene, Rome, Italy) supplemented with 10% heat-inactivated fetal bovine serum (FBS) (Aurogene, Rome, Italy), 1% L-glutamine (Biowest, Nuaillé, France), and 1% penicillin/streptomycin (Biowest, Nuaillé, France). Cells were cultured in a 5% CO_2_ humidified atmosphere at 37 °C. The cell lines were maintained in 75 cm^2^ flasks at a concentration of 2 × 10^6^ cells/mL and subcultured three times a week. Cells were treated with 200 μM temozolomide (TMZ; ThermoFisher Scientific, Waltham, MA, USA) for 48 h.

### 2.2. EV Isolation by Ultrafiltration Combined with Size Exclusion Chromatography (UF/SEC)

To isolate EVs from conditioned media, 10 × 10^6^ cells were seeded in 75 cm^2^ flasks and cultured for 48 h in DMEM 4.5 g/L glucose with 10% EV-depleted FBS (heat-inactivated FBS was centrifuged at 100,000× *g* at 4 °C for 18 h). To isolate EVs from TMZ-treated cells, 200 μM TMZ was added to culture media for 48 h of incubation. Conditioned cell culture media were sequentially centrifuged at 500× *g* (10 min), 800× *g* (10 min), and 2000× *g* (20 min) at room temperature (RT) to remove cells and debris. After, the total volume of 30 mL of media was loaded onto Amicon Ultra-15 Centrifugal Filter Units with Ultracel-10 membrane (molecular weight cut-off [MWCO] = 10 kDa; Merck Millipore, Billerica, MA, USA) and concentrated to 1.5 mL by repeated centrifugation at 4000× *g* (40 min, at 4 °C). Concentrated media was fractionated by SEC as described by Böing et al. with some modifications [18]. In brief, 1.5 mL of concentrated media was loaded into a 10 mL sepharose CL-2B column (GE Healthcare, Little Chalfont, UK) and the EV-rich protein-low fractions (8 to 10) were collected using PBS as eluent.

### 2.3. Cryogenic Electron Microscopy (Cryo-EM)

Cryo-EM samples of EVs derived from both untreated and TMZ-treated U87MG, U251MG, U373MG, and T98G cells were prepared on either Quantifoil R 1.2/1.3 Cu 300 (Quantifoil Micro Tools GmbH, Großlöbichau, Germany) or Lacey Carbon 200 mesh TH copper (Ted Pella, Redding, CA, USA) grids. The grids were first glow-discharged (20 mA, 45 s) in a PELCO easiGlow instrument (Ted Pella, Redding, CA, USA). Subsequently, in a Vitrobot Mark IV instrument (ThermoFisher Scientific, Waltham, MA, USA) a 4 μL aliquot of the undiluted aqueous solution of the sample was applied on the carbon side of the grid, which was then blotted for 6 s and plunge-frozen into the liquid ethane. EVs from three independent isolations were imaged and analyzed. Cryo-electron micrographs of vitrified samples were collected using a cryo-transmission electron microscope, Glacios (ThermoFisher Scientific, Waltham, MA, USA), equipped with a Falcon 3EC camera at an accelerating voltage of 200 kV. Grid mapping and image acquisition were performed using EPU software (version 2.12.1.2782REL, ThermoFisher Scientific, Waltham, MA, USA) at nominal magnifications of 155× and 13,500×, respectively. High-magnification images were recorded at 73,000× nominal magnification (0.197 nm pixel size) with a −3.0 µm defocus value. To minimize radiation damage during image acquisition, the accumulated total dose per image did not exceed 30 e^−^/Å^2^.

### 2.4. Morphometric Analysis

Image analysis was performed using ImageJ software Version 1.54r [19]. A total number of 150 images, including those at a magnification of 180×, 13,500×, and 73,000×, were analyzed. Only clearly visible vesicles with intact membrane bilayers were included in the analysis.

*Size*. The size was analyzed for all subtypes of EVs (SVs, DVs, and MVs). The size of each vesicle was taken as the diameter measured across a vesicle from one side of the outer membrane bilayer to the opposite side of it and expressed in nm. For DVs and MVs, the diameter of all the enclosing vesicles was measured regardless of EV concentricity.

*Circularity.* The area of each EV was automatically delineated using the “Analyze particles” tool with ImageJ. The circularity was assessed using “Shape descriptors” measurement and expressed on a scale of 0–1. EVs with values ranging between 1 and 0.95 were considered circular. Values below 0.95 indicated irregularly shaped EVs.

*Concentricity.* This parameter was only applicable to DVs and MVs, in which the position of inner EVs relative to enclosing EVs was analyzed. The distance (nm) between the membranes of inner EVs and the membranes of enclosing EVs was measured. If all the inner EVs were equidistant from the membrane of the enclosing vesicle, and thus sharing the same center, they were categorized as concentric. If one or more enclosed vesicles did not share the common center with the enclosing vesicle and were not equidistant from the membrane of the outer EV, they were classified as eccentric. Minor deviations (≤5 nm) were accepted due to image resolution and measurement variability.

*Membrane thickness*. Thickness was determined using line intensity profile analysis. The distance between the two electron-dense membrane leaflets was taken as the membrane thickness (distance, nm). The measurement of the thickness of the membrane bilayer of the enclosing EVs was taken for SVs, DVs, and MVs. For DVs and MVs the thickness of the lipid bilayer of enclosing vesicles (external membrane bilayer) was compared with the mean of all measurements of thickness of enclosed vesicles (internal membrane bilayers), regardless of their number in case of MVs.

*Electron density.* For electron density measurement, the images were inverted so that the highest value on the grayscale (255) indicated the highest electron density, and the lowest value (0) indicated the lowest electron density. The gray value (“Mean gray value” measurement in ImageJ) of the lumen of EVs was measured only for SVs. The gray value of the background was expressed as the mean of 5 random measurements in each image. The electron density was calculated as the difference between the measured gray value of EVs and the background.

### 2.5. Statistical Analysis

EV size distributions were assessed for normality using Shapiro–Wilk test (Graph-pad Prism 10 software, GraphPad Software, San Diego, CA, USA), which indicated non-normal distribution for all groups (*p* < 0.001). Therefore, unpaired nonparametric statistical analysis was performed. Mann–Whitney test was implemented to analyze EV size distributions, circularity, membrane thickness, and electron density. Values were expressed as mean ± SD; *p*-values < 0.05 were considered significant.

## 3. Results

Morphological characterization was performed for EVs isolated from four GBM cell lines: U87MG, U373MG, U251MG, and T98G. For each cell line, EVs were isolated by ultrafiltration combined with size-exclusion chromatography (UF/SEC) from untreated or TMZ-treated cells (200 μM TMZ, 48 h). The morphological parameters evaluated were size, shape, circularity, concentricity, membrane thickness, and electron density. In the representative images acquired by cryo-EM, EVs appear as particles heterogeneous in size and morphology, surrounded by one or more intact lipid bilayers (Supplementary Figures S1–S4).

### 3.1. Size

**Figure 1:**
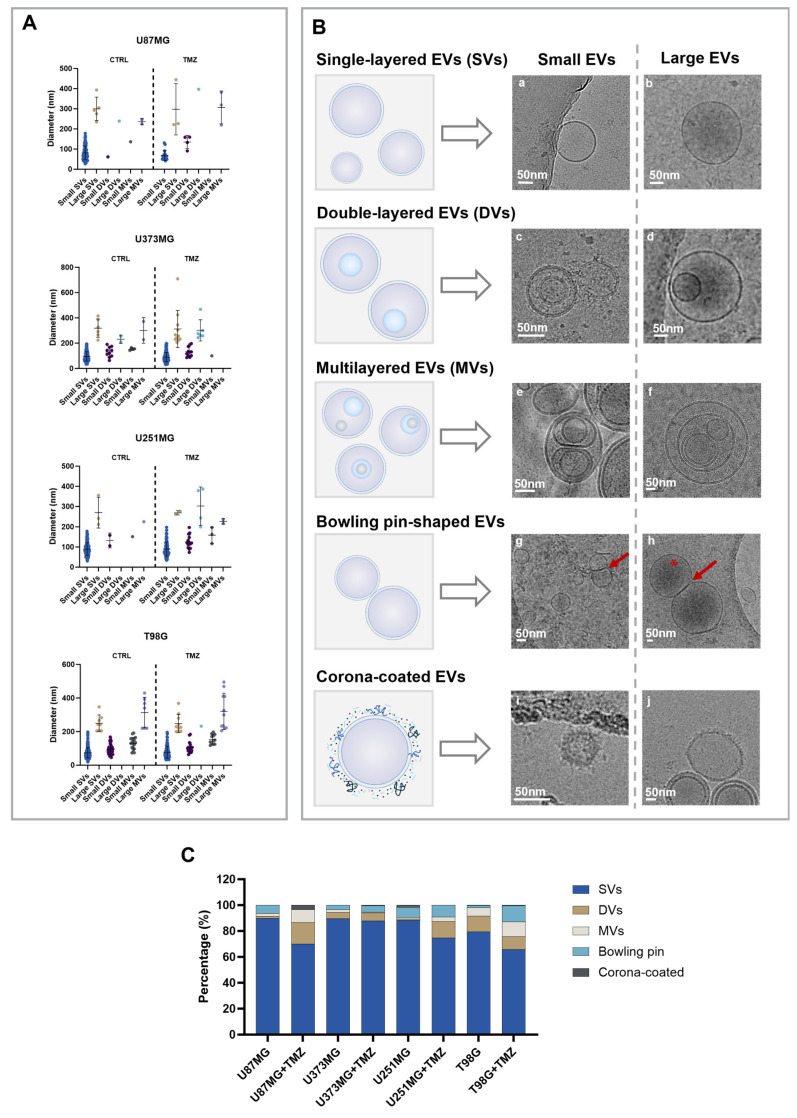
Cryo-EM analysis of the size and morphology of GBM-derived EVs. (**A**) Scatter dot plots showing the correlation between the size and the number of GBM-derived EVs isolated from untreated (CTRL) and TMZ-treated (TMZ) U87MG, U373MG, U251MG, and T98G cells for each EV subtype (SVs, DVs, and MVs). Results are presented as mean ± SD. (**B**) Representative cryo-EM images showing the structural heterogeneity of extracellular vesicles isolated from untreated and TMZ-treated GBM cell lines (scale bar 50 nm). The red arrows indicate bowling pin-shaped EVs; the red asterisk indicates EVs with electron-dense cargo. (**a**) U87MG, (**b**) U373MG, (**c**) U87MG + TMZ, (**d**) U373MG, (**e**) T98G + TMZ, (**f**) U87MG, (**g**) U251MG, (**h**) U373MG + TMZ, (**i**) U87MG + TMZ, (**j**) T98G. (**C**) Relative percentage of different morphological populations of EVs.

The diameter of EVs isolated from both untreated and TMZ-treated GBM cells, measured using ImageJ software, was used to classify them based on size, as small EVs (diameter < 200 nm) or large EVs (diameter > 200 nm), according to the MISEV2023 guidelines [2]. Approximately 200 vesicles were measured for each sample (Table 1). EV size was highly heterogeneous, with diameters ranging from 19.8 nm to 709 nm across vesicles derived from four cell lines (Figure 1A,B and Table 2). Regardless of the GBM cell line of origin, small EVs were the most abundant in both untreated and TMZ-treated conditions. In fact, only 6.2%, 4%, 2.3%, and 4% of the EV population were classified as large EVs in normal conditions, respectively, for U87MG, U373MG, U251MG, and T98G cells (Table 1). A reduction in the percentage of small EVs was observed in all experimental groups, with a higher extent in U87MG and T98G (17.9% and 8.5%, respectively). A slight reduction was also determined in U373MG- and U251MG-derived EVs, 2.6% and 3.9%, respectively (Table 1). For large EVs, in U87MG their proportion increased from 6.2% in the control group to 24.1% after TMZ treatment; the same trend, with a lesser extent, was observed in T98G (4% vs. 12.5%) and even less for U373MG and U251MG (4% vs. 6.6% and 2.3% vs. 6.2%, respectively).

### 3.2. Shape

In general, the morphology of EVs observed by cryo-EM was highly heterogeneous, with circular or irregularly shaped vesicles bound by one or multiple lipid bilayers. Both small and large EVs were categorized as SVs, DVs, or MVs (Figure 1A,B; Supplementary Figures S1–S4), depending on whether they contained one, two, or more than two lipid bilayers. Figure 1B shows representative images of SVs, DVs, and MVs, as well as bowling pin-shaped and corona-coated EVs, which represent less common morphologies in all conditions. Among the small EVs, in both untreated and TMZ-treated conditions, SVs were extremely numerous (Figure 1C). There was a clear tendency in the increment of EV size from SVs to DVs and MVs (Table 2). After TMZ treatment the percentage of SVs decreased (by more than 10%) while the percentages for DVs and MVs increased, except for U373MG-derived EVs (Figure 1C; Supplementary Figure S2). In terms of representativity of different subtypes, EVs isolated from U373MG cells were influenced the least by the treatment (Figure 1C; Supplementary Figure S2). In summary, SVs were the most abundant EVs across all analyzed samples, while changes in the relative proportions of SVs, DVs, and MVs were primarily influenced by the treatment. In contrast, the overall distribution and diversity of EV subtypes depended mainly on the specific cell line.

Finally, as reported above, at least two other morphologies were observed by cryo-EM. Firstly, the ‘bowling pin’-shaped EVs, which refer to two vesicles positioned in proximity or even sharing a merged lipid bilayer, and secondly, EVs coated by a thick peripheral corona layer. The latter morphology was rare (Figure 1B; Supplementary Figures S1–S4).

### 3.3. Circularity

**Figure 2:**
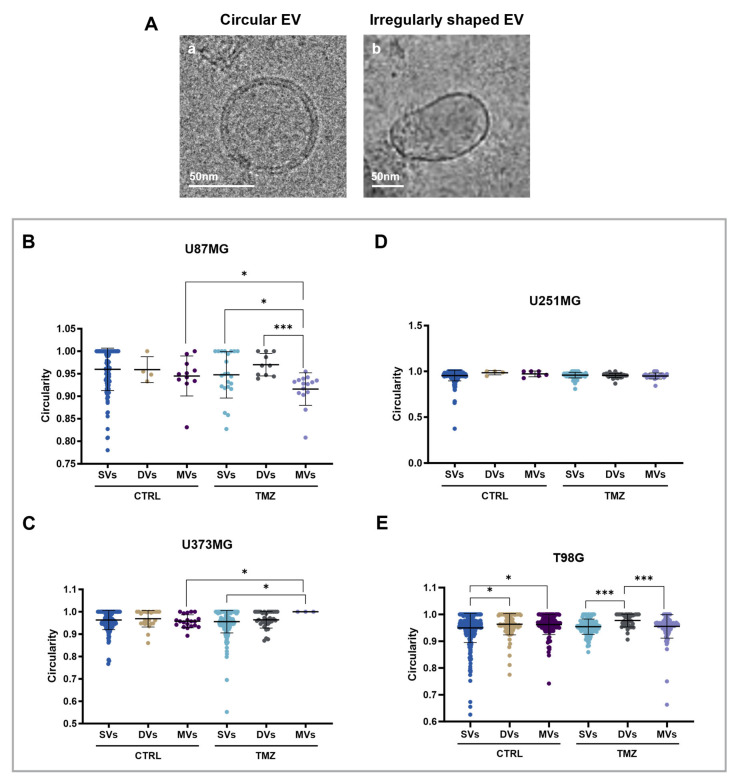
Cryo-EM analysis of circularity of GBM-derived EVs. (**A**) Representative cryo-EM images of circular and irregularly shaped SVs; scale bar 50 nm. (**a**) U87MG + TMZ, (**b**) U251MG. (**B**–**E**) Scatter dot plots representing the comparison of circularity of EVs derived from U87MG (**B**), U373MG (**C**), U251MG (**D**), and T98G (**E**) cell lines, untreated or TMZ-treated. Statistical analysis was conducted by the Mann–Whitney test. Data represents mean ± SD (* *p* < 0.05; *** *p* < 0.001).

All SVs, DVs, and MVs, irrespective of size, were categorized according to their circularity on a scale of 0–1. Values between 1 and 0.95 indicate circular EVs, while values below 0.95 indicate irregularly shaped EVs, i.e., elongated, tubular, or deformed vesicles. Figure 2A shows representative images of circular and irregularly shaped EVs. U87MG-derived MVs were the most irregular across all analyzed control groups (mean value of 0.945), and their circularity was the most affected by the treatment, with the mean value decreasing to 0.916 (Figure 2B; Supplementary Figure S1). Based on the mean values, SVs, DVs, and MVs from U373MG cells were classified as circular; however, this value for SVs and DVs decreased slightly after the treatment. All MVs from TMZ-treated U373MG cells were fully circular, with a mean value of 1.0 (Figure 2C; Supplementary Figure S2). Similarly to U373MG, U251MG-derived EVs were categorized as circular by their mean value and exhibited the least variability (Figure 2D; Supplementary Figure S3). SVs derived from T98G appeared irregularly shaped in the control group and circular in the TMZ-treated group (0.949 and 0.954, respectively) (Figure 2E; Supplementary Figure S4). No clear correlation in terms of circularity between subtypes of EVs or the treatment across all four cell lines was observed; but instead, it appeared to be an individual characteristic of each cell line.

### 3.4. Concentricity

**Figure 3:**
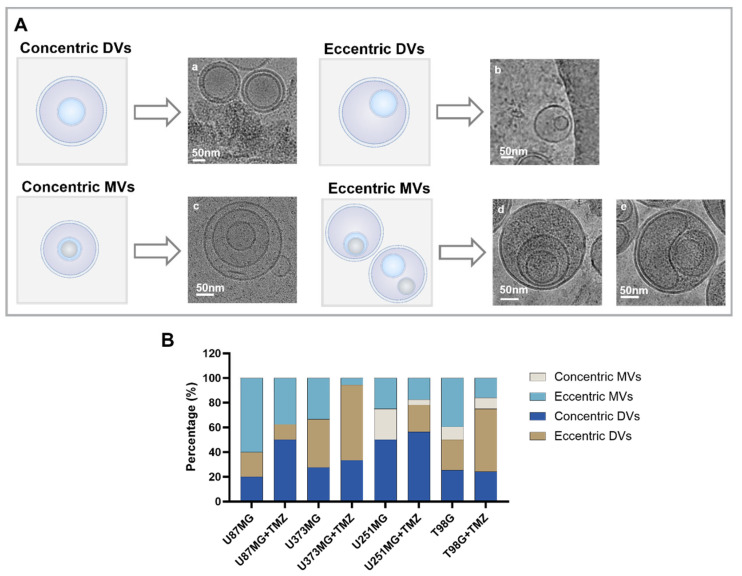
Cryo-EM analysis of concentricity of GBM-derived EVs. (**A**) Representative cryo-EM images demonstrating concentric and eccentric DVs and MVs (scale bar 50 nm). (**a**) T98G, (**b**) U373MG, (**c**) U251MG, (**d**,**e**) T98G + TMZ. (**B**) Relative percentage of concentric and eccentric DVs and MVs across four GBM cell lines.

For DVs and MVs, the concentricity of EVs was also analyzed. This parameter refers to the alignment of the centers of the inner and outer vesicles. Concentric EVs are those in which all enclosed vesicles share the same center with the enclosing vesicle. Conversely, when the inner EVs are shifted to either side within the enclosing vesicle, they are referred to as eccentric (Figure 3A). The percentage of eccentric MVs decreased in all four cell lines following treatment with TMZ (Figure 3B). Concentric MVs were only found in samples from the U251MG and T98G cell lines, and their number decreased with the treatment (Figure 3B; Supplementary Figures S3 and S4). The percentage of concentric DVs increased in the treated groups for U87MG, U373MG, and U251MG, whereas it remained unchanged in T98G. Eccentric DVs were more abundant in the treated groups, except for EVs derived from U87MG cells (Figure 3B; Supplementary Figure S1). Concentricity is more likely to be an individual characteristic of each cell line, but there are some similarities.

### 3.5. Membrane Thickness

**Figure 4:**
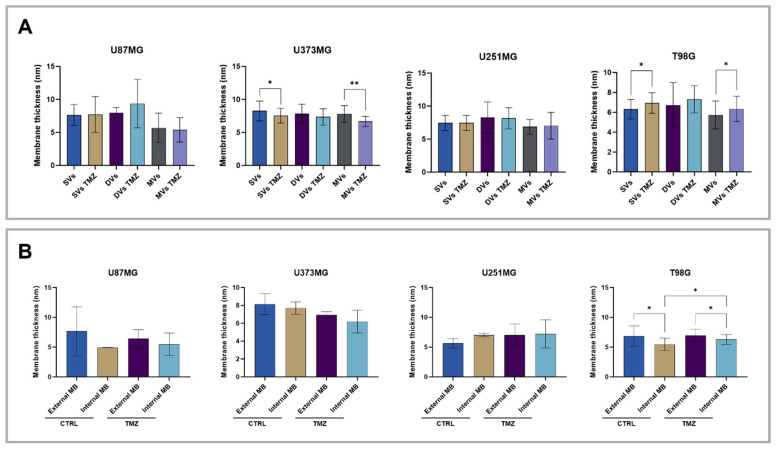
Analysis of membrane thickness in GBM-derived EVs acquired using cryo-EM. (**A**) Difference in external membranes thickness across SVs, DVs, and MVs in U87MG, U373MG, U251MG, and T98G cell lines (untreated and TMZ-treated). (**B**) Comparison between the thickness of external and internal membrane bilayers (MB) for DVs and MVs. Statistical analysis was conducted by the Mann–Whitney test. Data represents mean ± SD (* *p* < 0.05; ** *p* < 0.01).

Membrane thickness, another characteristic of membrane-enclosed particles, was examined in the different subtypes of EVs (SVs, DVs, MVs) derived from control and TMZ-treated U87MG, U373MG, U251MG, and T98G cells (Figure 4A). The thickness of the external membrane bilayer for all morphological groups, as well as the mean thickness of the internal membrane bilayers for DVs and MVs, was measured. No significant changes were observed for EVs derived from U87MG and U251MG cells. For EVs isolated from TMZ-treated U373MG cells, membrane thickness decreased for both SVs and MVs compared to control (Figure 4A). The opposite tendency was observed in T98G-derived EVs. Furthermore, for DVs and MVs, the thickness of the outer bilayer (external MB) vs the inner bilayers (internal MB; measured as the mean of the thickness of all inner layers) was analyzed to assess the influence of the treatment (Figure 4B). In general, except for U251MG, the internal membrane bilayers tended to be thinner than the external ones (Figure 4B). The thickness of internal membranes of T98G-derived EVs was significantly lower compared to the external bilayers in both the control and treated groups (Figure 4B). No significant variations were observed in the other experimental groups.

### 3.6. Electron Density

**Figure 5:**
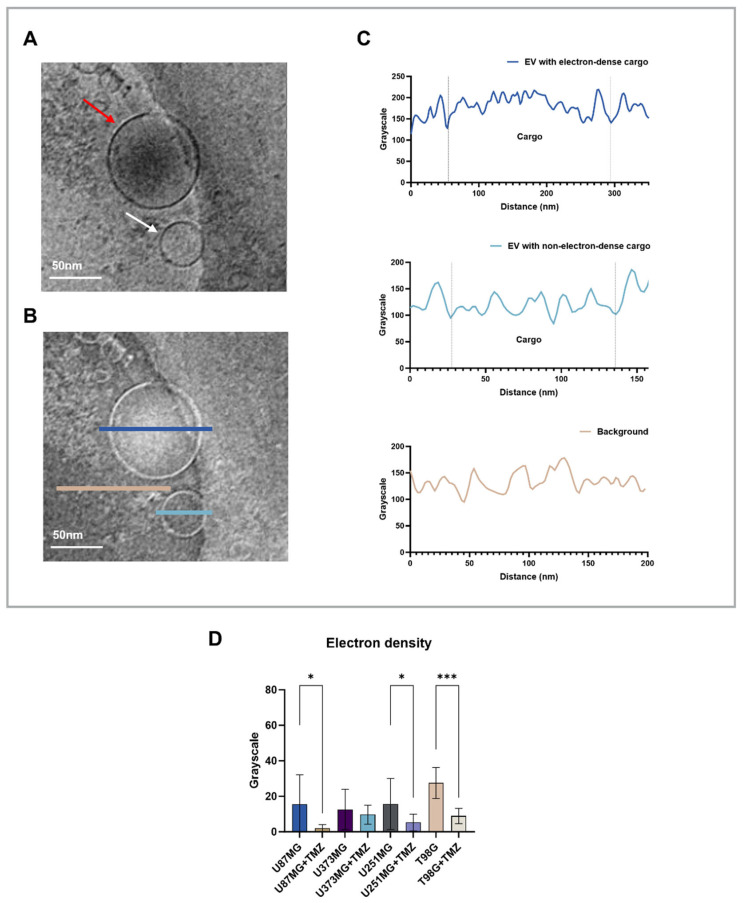
Analysis of electron density of GBM-derived EVs acquired using cryo-EM. (**A**) Representative cryo-EM image showing the difference in electron density between two SVs (U87MG CTRL) (scale bar 50 nm). Red arrows indicates EVs with a higher electron density; white arrows indicate EVs with a lower electron density. (**B**) Inverted cryo-EM image used for the generation of corresponding line profiles for electron density. Colored lines indicate the position of line scans used for profiling. (**C**) Electron density line profiles (grayscale) for EV with electron-dense cargo, EV with non-electron-dense cargo, and background. (**D**) Electron density of EVs measured in grayscale. Statistical analysis was conducted by the Mann–Whitney test. Data represents mean ± SD (* *p* < 0.05; *** *p* < 0.001).

Electron density, which refers to the difference in the measured grayscale value between EVs and the background, was analyzed for SVs only (Figure 5A). For the measurement of this parameter, cryo-EM images were inverted (Figure 5B). Figure 5C shows representative electron density line scans for EV with electron-dense cargo, EV with non-electron-dense cargo, and the background. For the control groups, the electron density of EVs was comparable between different cell lines, except for T98G, which was higher. A significant decrease in electron density of EVs was identified in three out of four cell lines after TMZ treatment compared to control (Figure 5D). The greatest decrease was observed in T98G-derived EVs (decreasing from 27.6 ± 8.71 to 8.96 ± 4.30), and the least in U373MG-derived EVs (decreasing from 12.6 ± 11.3 to 9.69 ± 5.38). However, although not significant, a decrease in electron density was also determined in EVs derived from TMZ-treated U373MG cells with respect to the control (Figure 5D).

## 4. Discussion

The present study, through cryo-EM analysis, reveals an extensive and previously underappreciated structural heterogeneity of EVs derived from GBM cells, which is further modulated by TMZ treatment. It has long been assumed that the diversity of EVs stems primarily from their cargo (including proteins, nucleic acids, and lipids) and size, leading to classification into small and large EVs [2]. However, growing evidence indicates that such diversity extends to functionally relevant morpho-biophysical properties [20]. Cryo-EM enables the visualization of the native structure of EVs, including the number of lipid bilayers, that cannot be observed with conventional techniques. Additionally, it facilitates the evaluation of the impact of disturbances to the extracellular environment, such as the addition of TMZ to the cell culture medium, on the quantity of produced EVs and morphological changes.

Our data shows that EVs isolated from four GBM cell lines (U87MG, U373MG, U251MG, and T98G) display high heterogeneity in terms of size, shape, number of different subtypes, membrane thickness, and electron density. Such heterogeneity of GBM-derived EVs reflects high variability among different cell lines as well as the cell-specific nature of their response to TMZ [21].

GBM is characterized by profound intra- and inter-tumoral heterogeneity, driven by genetic, epigenetic, and metabolic differences among tumor cells, as well as by their interaction with stromal and immune components [22]. In this context, EVs are key mediators of communication within the TME, contributing to proliferation, invasion, immune evasion, and therapy resistance [23]. Therefore, the structural diversity observed in our study is unlikely to be a passive feature, but rather reflects functional specialization of EV subpopulations, potentially linked to distinct roles in tumor progression. Indeed, EV heterogeneity has been previously described; for instance, EVs derived from GSCs of proneural or mesenchymal subtypes exhibit distinct proteomic profiles and, as a result, differential effects on recipient cells [24]. A central finding of this work is the cell line-specific effect of TMZ on EV morphology and production. Given the heterogeneity of GBM and their specific interactions with the microenvironment, chemotherapy may not act uniformly but instead reprograms EV biogenesis and release in a context-dependent manner, contributing to adaptive responses within the TME [8,9,10,25].

Significant variability between GBM-derived EVs cannot be attributed to the isolation procedure, since all EV samples were prepared using the UF/SEC from conditioned cell culture media of both untreated and TMZ-treated cells. This method is considered the most reproducible and the least aggressive, thus creating fewer artifacts, as reported in studies involving isolation from various matrices. In particular, the UF/SEC procedure is a less damaging method of EV isolation compared to ultracentrifugation [26]. Moreover, comparison of isolation methods such as SEC, differential ultracentrifugation (dUC), and density gradient (DG) showed similar distribution of spherical and tubular EVs; however, some particle parameters differed based on the technique used [27]. Thus, high heterogeneity reflects differences in the cell of origin, biogenesis pathways, and environmental conditions, ultimately influencing their biological activity [28]. In this context, our findings demonstrate that morphological heterogeneity is an intrinsic and biologically meaningful dimension of EV diversity, with structural differences potentially related to cargo composition and function. This aligns with recent reviews emphasizing that EV heterogeneity arises from the integration of multiple levels, including cellular heterogeneity, vesiculation mechanisms, and microenvironmental modulation [29,30].

All particles with a clearly visible membrane bilayer and translucent lumen, visualized with cryo-EM, were identified as EVs. Neither lipid droplets, distinguished from EVs by their lipid monolayer, nor protein aggregates, which lack a lipid membrane, were observed. It is worth mentioning that size alone is insufficient to distinguish between protein aggregates, lipid droplets, and EVs [2].

Depending on the number of lipid bilayers, EVs were mainly categorized into three subtypes: SVs, with one lipid bilayer; DVs, with two lipid bilayers; and MVs, with more than two lipid bilayers. Importantly, our data indicate that TMZ treatment is associated with a shift in EV structural organization, characterized by a decrease in SVs (more than 10% in U87MG, U251MG, and T98G; and 2% in U373MG) and an increase in DVs and MVs. This suggests that chemotherapy affects not only EV quantity, but also their biogenesis and membrane architecture. A plausible mechanistic explanation lies in TMZ-induced alterations in lipid metabolism and membrane composition, which are known to regulate membrane curvature, rigidity, and vesicle formation [31]. Indeed, lipid metabolic rewiring is a hallmark of GBM and has been directly linked to changes in cell morphology and membrane dynamics, with implications for vesicle formation and release [32]. Consistently, previous reports [33] confirm that the morphological changes observed here reflect underlying TMZ-induced biochemical remodeling. The presence and increased abundance of MVs following TMZ treatment represents one of the most intriguing findings of this study. Although their origin is not fully understood, several mechanisms have been proposed, including repeated intraluminal vesicle formation within multivesicular bodies, the encapsulation of smaller vesicles during budding, or vesicle–vesicle fusion events, particularly under stress, and the effects of physical conditions like pH or pressure [30,34]. Another possible explanation for the observed alterations in the number of EV subtypes is the effect of TMZ on exosome biogenesis, which may lead to a decrease in the production of SVs. In GBM, TMZ induces autophagy, which represents one of the mechanisms of chemotherapy resistance [35,36]. Exosome biogenesis and autophagy are interconnected [37], as evidenced by the formation of amphisomes following the fusion of MVBs and autophagosomes during autophagy activation. This leads to reduced fusion of MVBs with the plasma membrane and subsequently decreases EV release [38]. Thus, cell stress and autophagy induced by TMZ may influence EV biogenesis through MVBs formation, maturation, and fusion, resulting in fewer SVs. Notably, increases in multilayered EVs have been reported under prolonged stress and in pathological contexts, supporting their biological relevance rather than representing an artifact of isolation [16,34,39]. Multilayered vesicles have been identified in diverse conditioned cell culture media (including GBM models), as well as in cerebrospinal fluid, ejaculates, and plasma [12,15,16,17,40,41].

TMZ treatment may favor the formation of multilayered EVs through different potential mechanisms. TMZ is known to induce oxidative stress, DNA damage responses, autophagy activation, and profound metabolic rewiring in GBM cells [35,42,43,44]. Since EV biogenesis is tightly linked to endosomal maturation and membrane remodeling, TMZ stress-induced perturbations of these pathways may promote repeated intraluminal budding, defective MVB maturation, vesicle encapsulation events, or abnormal membrane fusion processes, ultimately generating multilayered vesicles [30]. In addition, TMZ-induced alterations in lipid metabolism may directly influence membrane curvature, rigidity, and bilayer organization. Such lipid remodeling could facilitate the stabilization of multilamellar structures and alter EV membrane dynamics.

From a functional perspective, EV structural complexity may confer specific biological advantages. Multiple membrane layers could enhance cargo protection, enable compartmentalization of bioactive molecules, or support controlled, sequential release mechanisms, thereby increasing their efficiency in intercellular communication [45]. The proportion of SVs compared to MVs was shown to be higher in multiple studies [16,46]. In contrast, MVs isolated from gastric juice dominated the EV population, accounting for 56%, which may serve to protect cargo in the presence of digestive enzymes and under acidic pH [45]. In the context of GBM, where EVs actively shape the TME, such properties may contribute to more robust signaling, increased resistance to degradation, and improved delivery of oncogenic cargo, ultimately promoting tumor progression and therapeutic resistance. Additionally, the observed decrease in electron density following TMZ treatment suggests alterations in vesicular cargo composition, further supporting a link between structural remodeling and functional output. The presence of EVs with electron-dense cargo was noticed but not fully explained in published articles [41,47].

Although some artifactual contributions related to isolation and storage cannot be entirely excluded, the observation of MVs in minimally processed samples supports their classification as a bona fide vesicle subtype [12]. It is also worth noting that complementary approaches, such as atomic force microscopy (AFM), can help distinguish single-, double-, and multilayered vesicles based on their mechanical properties by quantifying penetration events during nanoindentation experiments [48].

Only a small subset of EVs, no more than 8% of the total population, exhibits an unusual shape, which was identified as bowling pin-shaped EVs. Such morphology may arise from the fusion of two lipid bilayers of the vesicles in proximity. Membrane fusion is not uncommon and occurs during processes such as EV internalization and endosomal escape [49,50]. Fusion between EVs and liposomes, namely large unilamellar vesicles (LUVs) that mimic the lipid composition of the plasma membrane, has been visualized using cryo-EM. This process is dependent on pH acidification and protein composition [51]. Given the distinct origins of exosomes and ectosomes, differences in membrane composition may promote fusion events and explain the presence of bowling pin morphology.

An even smaller proportion of EVs were coated with a protein corona, which is formed through adsorption of proteins onto their surface in protein-rich environments. In this study, the low proportion of corona-coated EVs may be explained by the use of depleted cell culture media, which contains lower protein levels than biological fluids such as plasma [52].

GBM-derived EVs also exhibited significant heterogeneity in circularity, concentricity, and membrane thickness, parameters that are rarely investigated but provide important insight into vesicle organization and dynamics. It can only be speculated that the differences in these EV characteristics may be caused by the change in lipid composition of membranes. Enrichment in saturated lipids with longer fatty acid chains could lead to a more rigid structure of vesicles’ membranes, while the opposite could lead to more fluid membranes [53,54]. Membrane-associated proteins such as BAR and phospholipids play a role in shaping membranes and potentially affect curvature, resulting in heterogeneity of EV morphologies from spherical to irregularly shaped [27,55]. Cryo-EM provides a snapshot of vesicles and does not offer insight into their dynamics; thus, in DVs and MVs, smaller inner EVs may move inside the enclosing vesicles and change their concentricity. The absence of a uniform trend across cell lines suggests that these characteristics are cell-specific and likely governed by intrinsic regulatory mechanisms, including cytoskeletal organization and membrane composition. Moreover, concentric versus eccentric organization in multilayered vesicles may reflect different stages of vesicle formation or post-release remodeling, potentially involving fusion events or internal rearrangements.

Taken together, our findings support a model in which EV heterogeneity is not solely a reflection of stochastic variability but rather the result of integrated processes involving cellular state, microenvironmental conditions, and metabolic regulation. Morphological diversity, including variations in bilayer number, membrane organization, and vesicle geometry, should therefore be considered a key determinant of EV function, rather than a secondary descriptive feature.

## 5. Conclusions

Overall, our data provides a comprehensive analysis of the morphological characteristics of GBM-derived EVs. To our knowledge, this is the most extensive research on EVs derived from GBM cells and the only one that shows the effects of TMZ on the nanostructural characteristics of EVs. Thus, EV morphology should not be underestimated as it may play a significant role in cell signaling. A possible differential role among different EV subtypes, with distinct clinical relevance, may be hypothesized. However, to confirm these functional roles, technical improvements for the selective isolation of SVs, DVs, and MVs will be required.

The use of EVs in drug delivery is a promising therapeutic concept. Successful loading of GBM-derived EVs with TMZ is reported in the literature [56,57], where also anti-tumoral effects have been shown. However, the application of cancer cell-derived EVs may pose a risk since they carry oncogenic cargo [11,58]. To avoid these potential implications, it may be more advantageous to develop delivery systems based on non-cancerous cell-derived EVs [59], plant-derived EVs [60], biomimetic microparticles [61], or nanoparticles [62].

Our research further confirms that cryo-EM alone is a unique method for high-throughput morphological characterization and a valuable tool for strengthening functional studies.

## Figures and Tables

**Table 1:** Proportion of small and large EVs (%) in control (CTRL) and TMZ-treated (TMZ) groups of U87MG, U373MG, U251MG, and T98G cells and the reduction in the amount of small EVs in the TMZ-treated group compared to the control (Δ Small EVs).

Cell Line	Condition	Small EVs (%)	Large EVs (%)	Δ Small EVs (TMZ/CTRL)
U87MG	CTRL	93.8	6.2	−17.9%
	TMZ	75.9	24.1	
U373MG	CTRL	96.0	4.0	−2.6%
	TMZ	93.4	6.6	
U251MG	CTRL	97.7	2.3	−3.9%
	TMZ	93.8	6.2	
T98G	CTRL	96.0	4.0	−8.5%
	TMZ	87.5	12.5	

**Table 2:** Percentage and average size in nm (Mean ± SD) of different morphological subtypes (SVs, DVs, and MVs) of small and large GBM-derived EVs in control (CTRL) and TMZ-treated (TMZ) U87MG, U373MG, U251MG, and T98G cells. Values marked with an asterisk (*) represent single measurements.

	CTRL			TMZ		
		Percentage	Size		Percentage	Size
U87MG						
SVs	Small	92.31	80.99 ± 34.25	Small	62.07	68.12 ± 24.32
	Large	3.85	299.9 ± 58.37	Large	10.34	297.6 ± 127.3
DVs	Small	0.77	61.60 *	Small	13.79	135.3 ± 32.08
	Large	0.77	238.9 *	Large	3.45	396.7 *
MVs	Small	0.77	136.5 *	Small	0.00	-
	Large	1.54	236.3 ± 15.21	Large	10.34	306.7 ± 81.95
U373MG						
SVs	Small	90.51	95.03 ± 37.02	Small	88.80	89.02 ± 37.38
	Large	2.37	318.2 ± 72.62	Large	4.25	312.3 ± 146.3
DVs	Small	3.95	129.8 ± 40.90	Small	4.25	131.3 ± 39.10
	Large	0.79	231.2 ± 33.26	Large	3.32	300.9 ± 83.86
MVs	Small	1.58	153.2 ± 9.186	Small	0.39	99.71 *
	Large	0.79	300.1 ± 101.3	Large	0.00	-
U251MG						
SVs	Small	95.93	89.96 ± 31.54	Small	80.62	90.19 ± 35.01
	Large	1.74	269.9 ± 75.79	Large	1.55	268.6 ± 10.34
DVs	Small	1.16	132.0 ± 36.82	Small	10.85	125.4 ± 34.39
	Large	0.00	-	Large	3.10	302.3 ± 94.54
MVs	Small	0.58	151.5 *	Small	2.33	158.6 ± 40.46
	Large	0.58	225.7 *	Large	1.55	226.7 ± 13.32
T98G						
SVs	Small	78.65	73.74 ± 29.88	Small	69.89	77.43 ± 35.23
	Large	2.16	249.8 ± 49.26	Large	5.68	247.9 ± 54.61
DVs	Small	12.43	93.95 ± 29.08	Small	10.80	104.6 ± 31.87
	Large	0.00	-	Large	0.57	232.4 *
MVs	Small	4.86	130.3 ± 35.74	Small	6.82	153.1 ± 28.53
	Large	1.89	313.2 ± 90.93	Large	6.25	320.6 ± 108.1

## Data Availability

The original contributions presented in this study are included in the article/Supplementary Materials. Further inquiries can be directed to the corresponding author.

## Supplementary Materials

The following supporting information can be downloaded at https://www.mdpi.com/article/10.3390/nano16110685/s1: Figure S1. Representative micrographs of different types of EVs identified in U87MG cell line. Figure S2. Representative micrographs of different types of EVs identified in U373MG cell line. Figure S3. Representative micrographs of different types of EVs identified in U251MG cell line. Figure S4. Representative micrographs of different types of EVs identified in T98G cell line. All uncropped images are included in the supplementary materials.
