# Supplementary material for: Cryogenic Electron Microscopy of Extracellular Vesicles from Temozolomide-Treated Glioblastoma Cells Reveals Great Morphological Heterogeneity
Source: Nanomaterials (Basel). 2026 Jun 1;16(11):685. doi: 10.3390/nano16110685 (PMC13257892; doi:10.3390/nano16110685)
Supplement: Supplementary file 1 [file nanomaterials-16-00685-s001.zip › Cryo paper uncropped images.pdf]

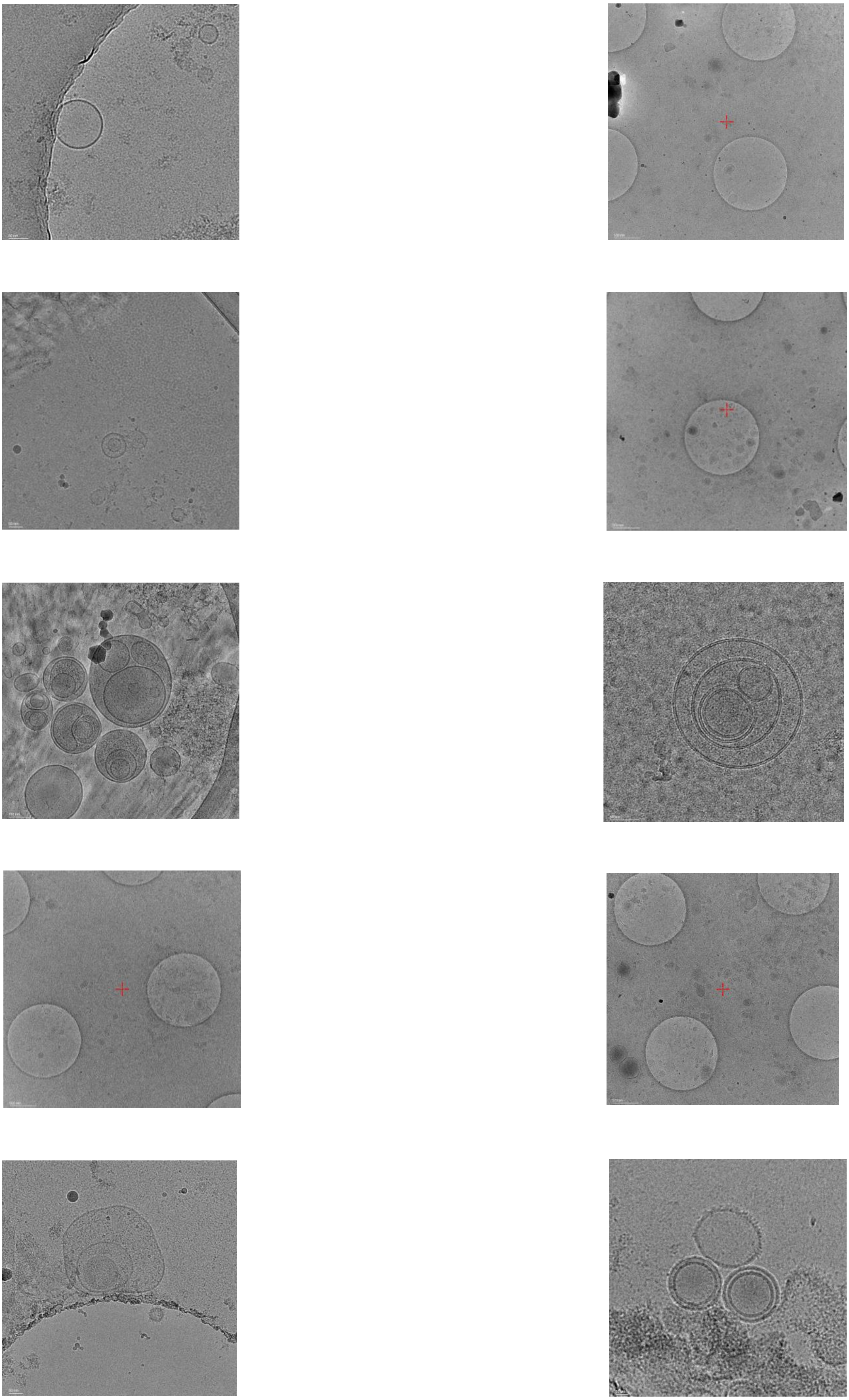

Figure 1.

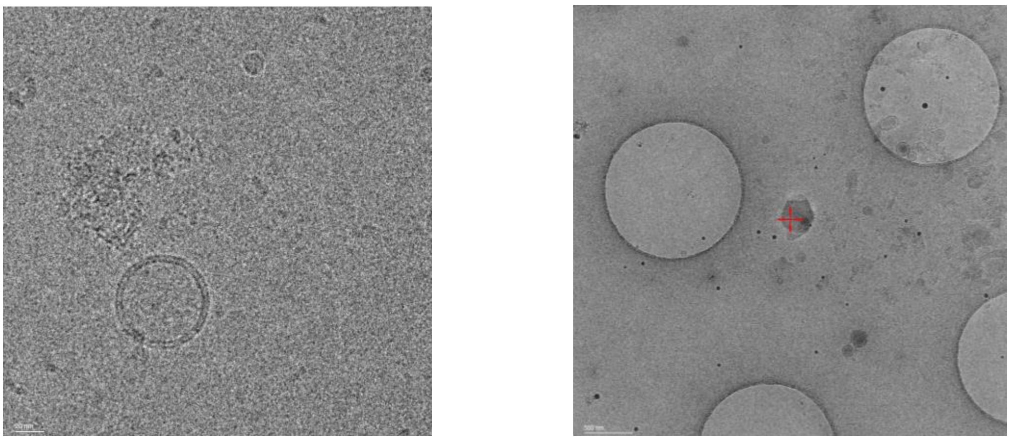

Figure 2.

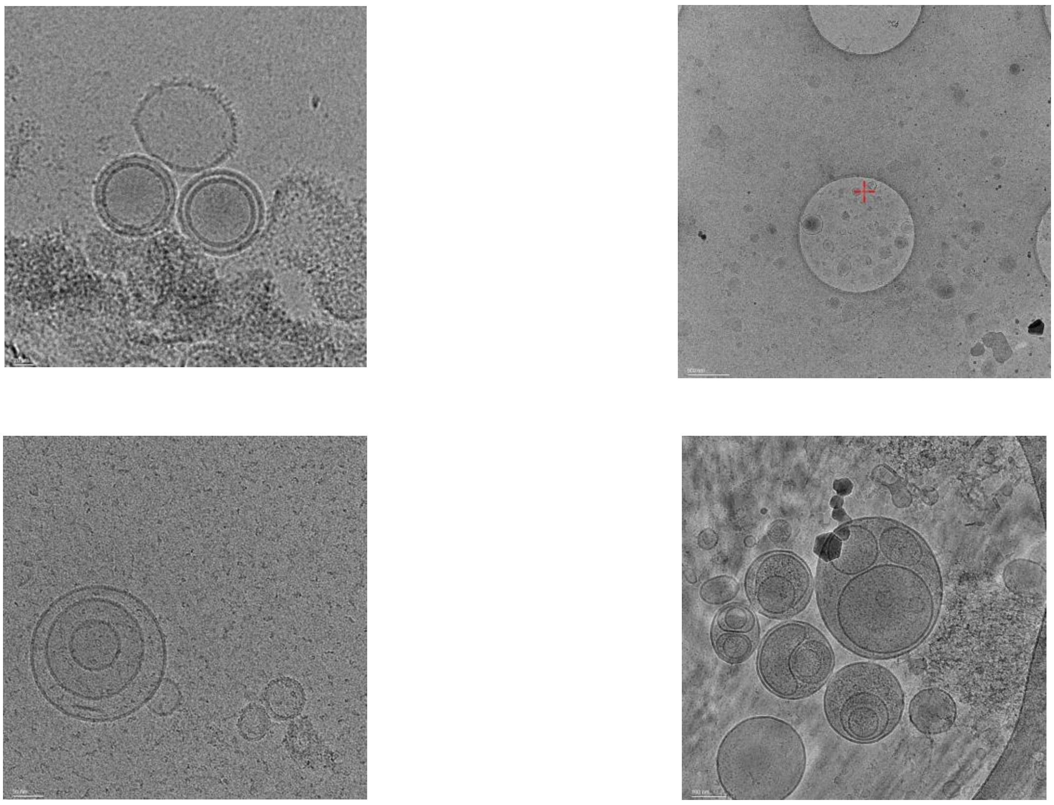

Figure 3.

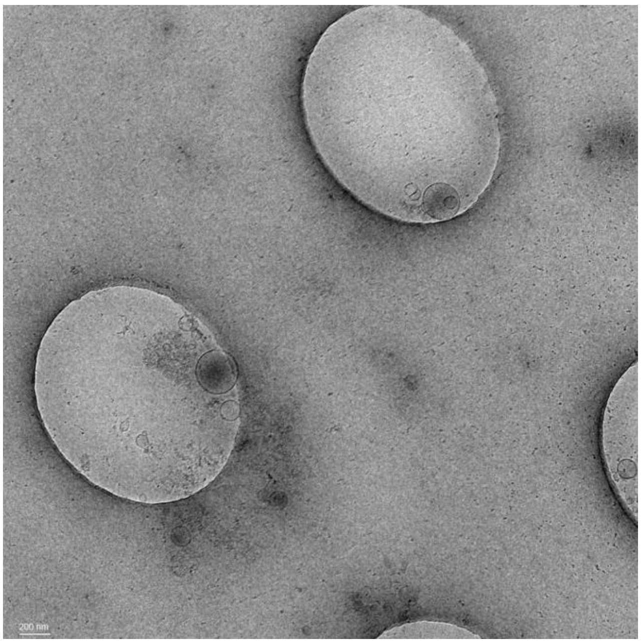

Figure 5.

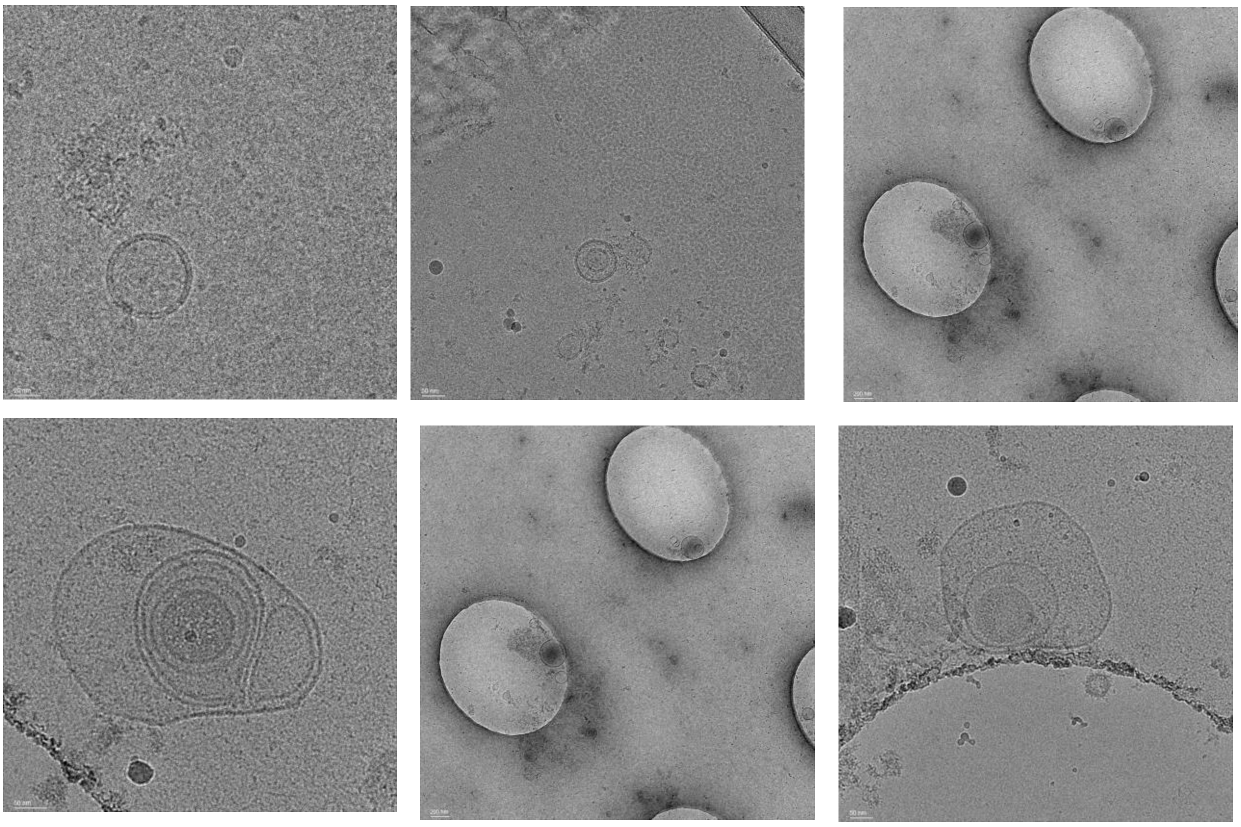

Supplementary figure S1.

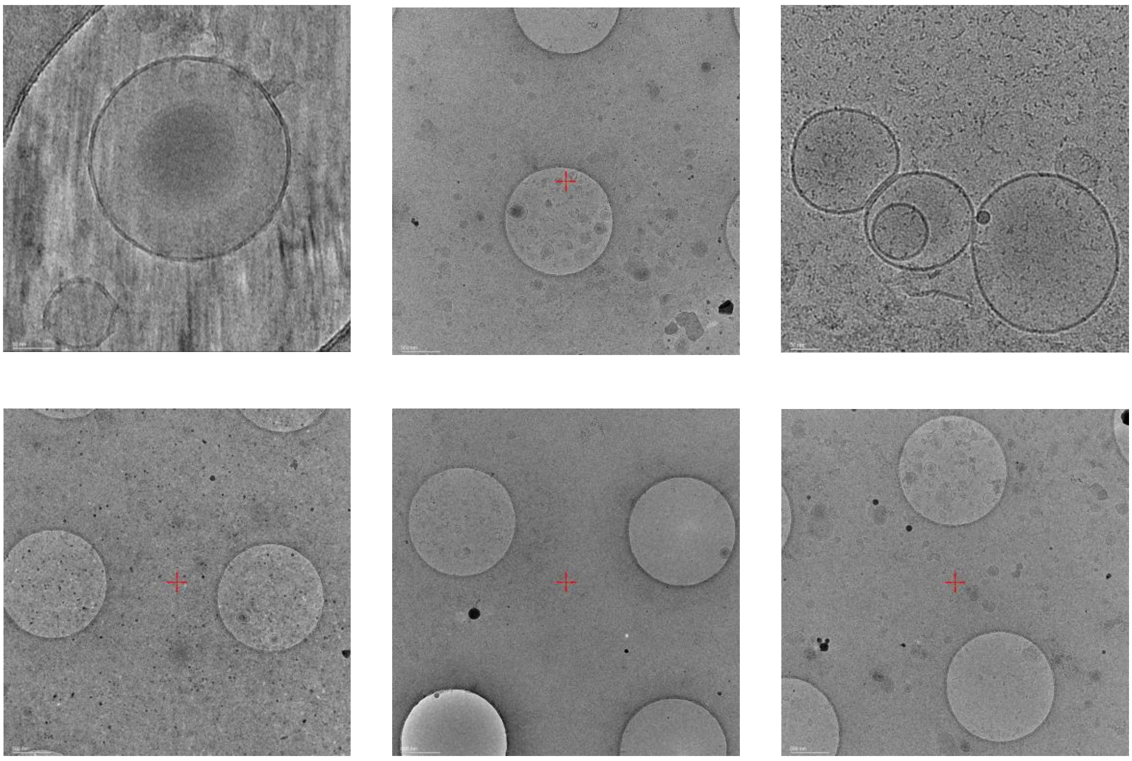

Supplementary figure S2.

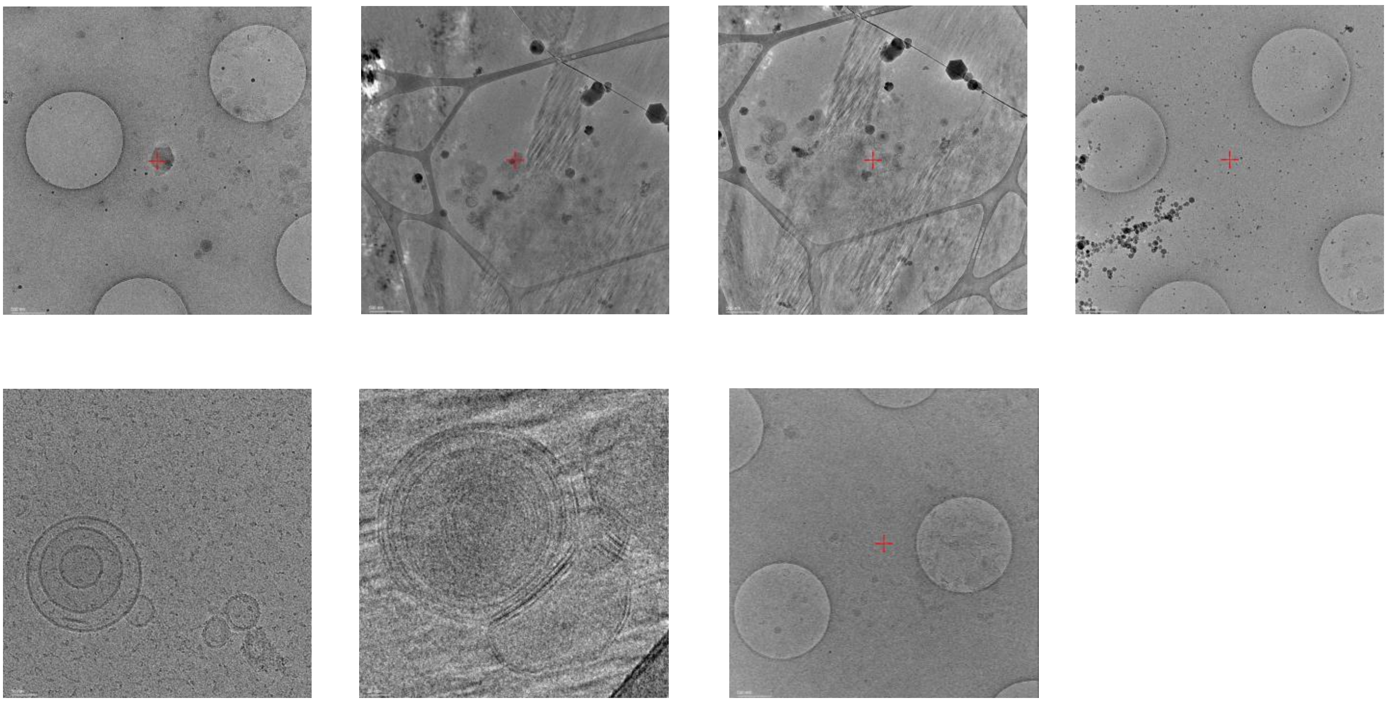

Supplementary figure S3.

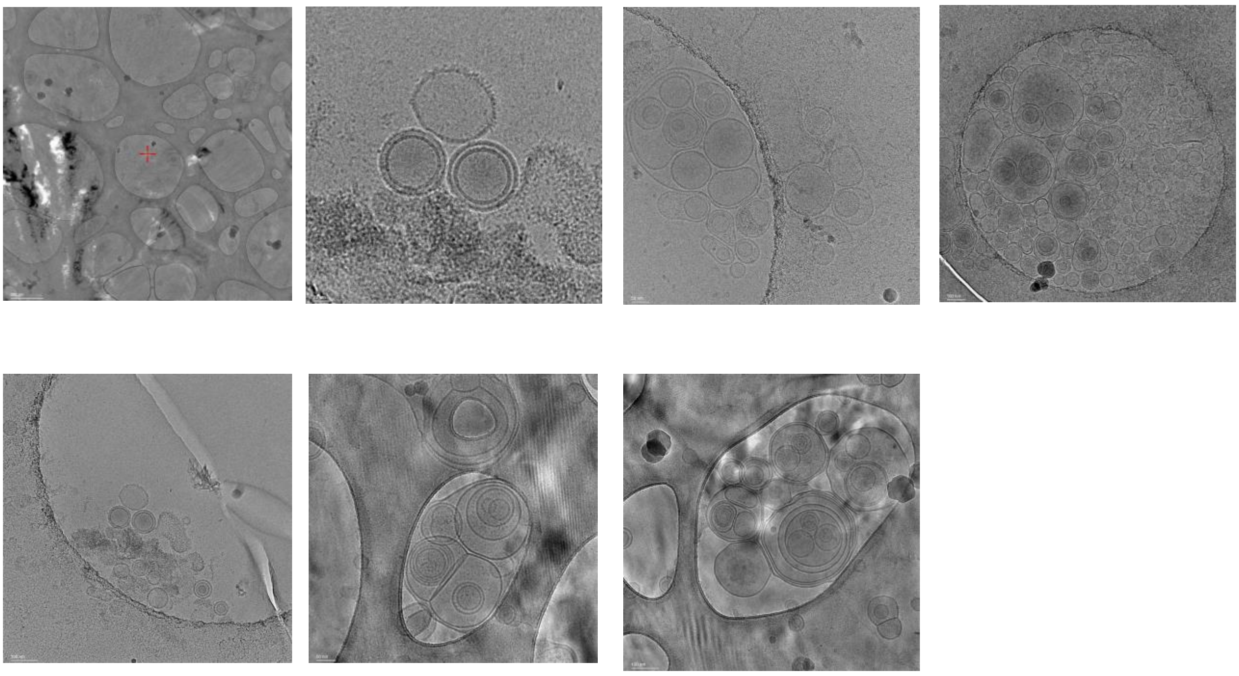

Supplementary figure S4.
