# Supplementary material for: Cryogenic Electron Microscopy of Extracellular Vesicles from Temozolomide-Treated Glioblastoma Cells Reveals Great Morphological Heterogeneity
Source: Nanomaterials (Basel). 2026 Jun 1;16(11):685. doi: 10.3390/nano16110685 (PMC13257892; doi:10.3390/nano16110685)
Supplement: Supplementary file 1 [file nanomaterials-16-00685-s001.zip › Supplementary figures.pdf]

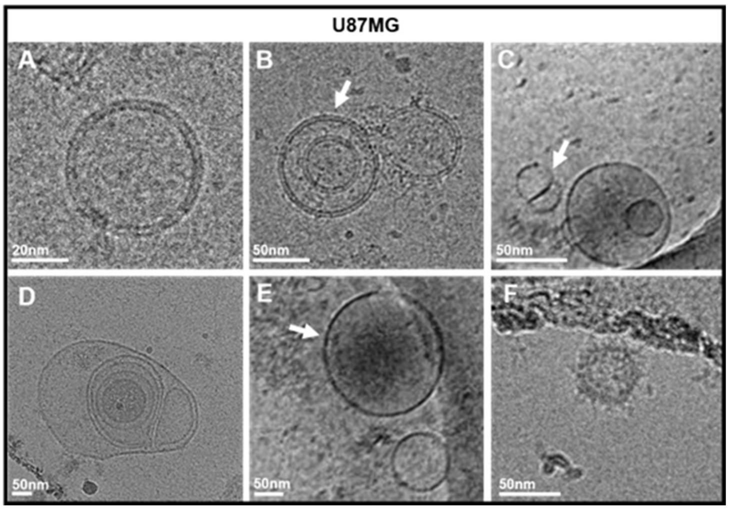

Supplementary Figure S1. Representative micrographs of different types of EVs identified in U87MG cell line. (A) Single vesicle (U87MG+TMZ); (B) Concentric double vesicle (white arrow; U87MG+TMZ); (C) Eccentric double vesicle; bowling pin-shaped vesicle (white arrow; U87MG CTRL); (D) Eccentric multilayer vesicle (U87MG+TMZ); (E) Single vesicle with electron dense cargo (white arrow; U87MG CTRL); (F) Corona-coated single vesicle (U87MG+TMZ).

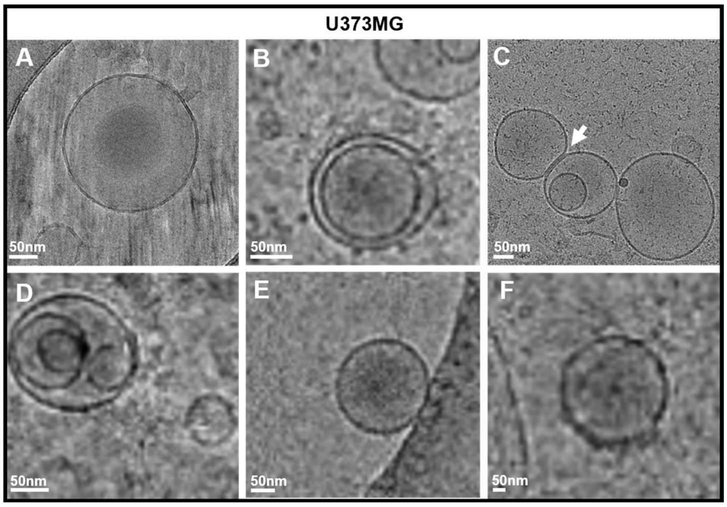

Supplementary Figure S2. Representative micrographs of different types of EVs identified in U373MG cell line. (A) Single vesicle (U373MG+TMZ); (B) Concentric double vesicle (U373MG CTRL); (C) Eccentric double vesicle; bowling pin-shaped vesicle (white arrow; U373MG CTRL); (D) Eccentric multilayer vesicle (U373MG CTRL); (E) Single vesicle with electron dense cargo (U373MG CTRL); (F) Corona-coated single vesicle (U373MG+TMZ).

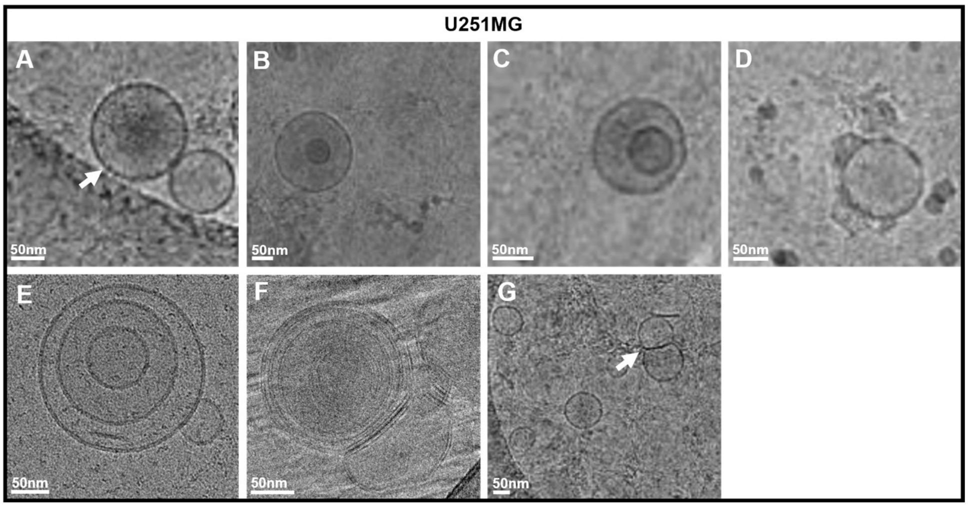

Supplementary Figure S3. Representative micrographs of different types of EVs identified in U251MG cell line. (A) Single vesicle and vesicle with electron dense cargo (white arrow; U251MG CTRL); (B) Concentric double vesicle (U251MG CTRL); (C) Eccentric double vesicle (U251MG+TMZ); (D) Corona-coated single vesicle (U251MG CTRL); (E) Concentric multilayer vesicle (U251MG CTRL); (F) Eccentric multilayer vesicle (U251MG+TMZ); (G) Bowling pin-shaped vesicle (white arrow; U251MG CTRL).

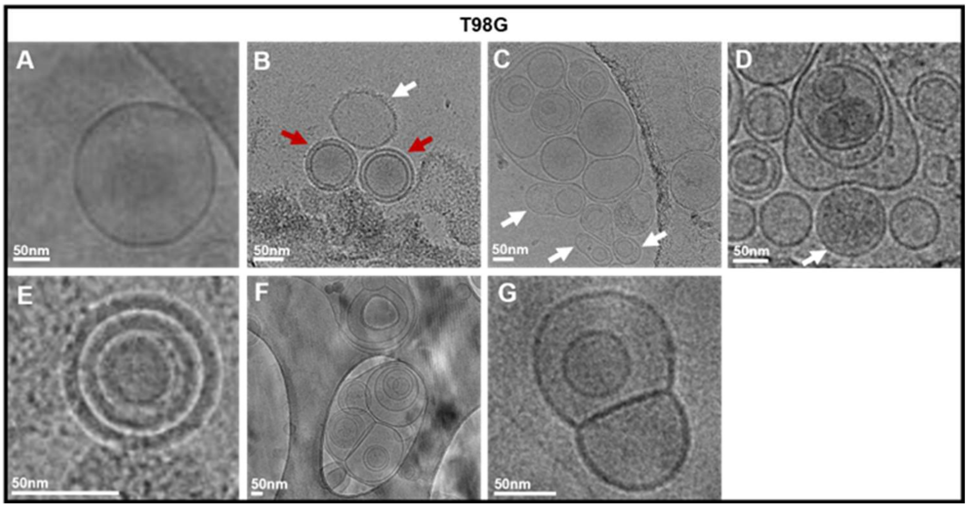

Supplementary Figure S4. Representative micrographs of different types of EVs identified in T98G cell line. (A) Single vesicle (T98G+TMZ); (B) Concentric double vesicle (red arrows) and corona-coated vesicle (white arrow; T98G CTRL); (C) Eccentric double vesicles (white arrows; T98G CTRL); (D) Single vesicle with electron dense cargo (white arrow; T98G CTRL); (E) Concentric multilayer vesicle (T98G+TMZ); (F) Eccentric multilayer vesicles (T98G+TMZ); (G) Bowling pin-shaped vesicle (T98G CTRL).
